# A Mouse with an *N*-Ethyl-*N*-Nitrosourea (ENU) Induced Trp589Arg *Galnt3* Mutation Represents a Model for Hyperphosphataemic Familial Tumoural Calcinosis

**DOI:** 10.1371/journal.pone.0043205

**Published:** 2012-08-13

**Authors:** Christopher T. Esapa, Rosie A. Head, Jeshmi Jeyabalan, Holly Evans, Tertius A. Hough, Michael T. Cheeseman, Eugene G. McNally, Andrew J. Carr, Gethin P. Thomas, Matthew A. Brown, Peter I. Croucher, Steve D. M. Brown, Roger D. Cox, Rajesh V. Thakker

**Affiliations:** 1 Academic Endocrine Unit, Nuffield Department of Clinical Medicine, University of Oxford, Oxford Centre for Diabetes, Endocrinology and Metabolism, Churchill Hospital, Oxford, United Kingdom; 2 Medical Research Council (MRC) Mammalian Genetics Unit and Mary Lyon Centre, MRC Harwell, Harwell Science and Innovation Campus, United Kingdom; 3 The Mellanby Centre for Bone Research, Department of Human Metabolism, University of Sheffield, Sheffield, United Kingdom; 4 Department of Radiology, Nuffield Orthopaedic Centre and Nuffield Department of Orthopaedics, Rheumatology and Musculoskeletal Sciences, University of Oxford, United Kingdom; 5 NIHR Biomedical Research Unit, Nuffield Department of Orthopaedics, Rheumatology and Musculoskeletal Sciences, University of Oxford, United Kingdom; 6 University of Queensland Diamantina Institute, Princess Alexandra Hospital, University of Queensland, Australia; 7 Garvan Institute for Medical Research, Sydney, Australia; Institute of Environmental Health, United States of America

## Abstract

Mutations of UDP-N-acetyl-alpha-D-galactosamine polypeptide N-acetyl galactosaminyl transferase 3 (GALNT3) result in familial tumoural calcinosis (FTC) and the hyperostosis-hyperphosphataemia syndrome (HHS), which are autosomal recessive disorders characterised by soft-tissue calcification and hyperphosphataemia. To facilitate *in vivo* studies of these heritable disorders of phosphate homeostasis, we embarked on establishing a mouse model by assessing progeny of mice treated with the chemical mutagen *N*-ethyl-*N*-nitrosourea (ENU), and identified a mutant mouse, TCAL, with autosomal recessive inheritance of ectopic calcification, which involved multiple tissues, and hyperphosphataemia; the phenotype was designated TCAL and the locus, *Tcal*. TCAL males were infertile with loss of Sertoli cells and spermatozoa, and increased testicular apoptosis. Genetic mapping localized *Tcal* to chromosome 2 (62.64–71.11 Mb) which contained the *Galnt3*. DNA sequence analysis identified a Galnt3 missense mutation (Trp589Arg) in TCAL mice. Transient transfection of wild-type and mutant *Galnt3*-enhanced green fluorescent protein (EGFP) constructs in COS-7 cells revealed endoplasmic reticulum retention of the Trp589Arg mutant and Western blot analysis of kidney homogenates demonstrated defective glycosylation of Galnt3 in *Tcal/Tcal* mice. *Tcal/Tcal* mice had normal plasma calcium and parathyroid hormone concentrations; decreased alkaline phosphatase activity and intact Fgf23 concentrations; and elevation of circulating 1,25-dihydroxyvitamin D. Quantitative reverse transcriptase-PCR (qRT-PCR) revealed that *Tcal/Tcal* mice had increased expression of *Galnt3* and *Fgf23* in bone, but that renal expression of *Klotho*, 25-hydroxyvitamin D-1α-hydroxylase (*Cyp27b1*), and the sodium-phosphate co-transporters type-IIa and -IIc was similar to that in wild-type mice. Thus, TCAL mice have the phenotypic features of FTC and HHS, and provide a model for these disorders of phosphate metabolism.

## Introduction

**Figure 1:**
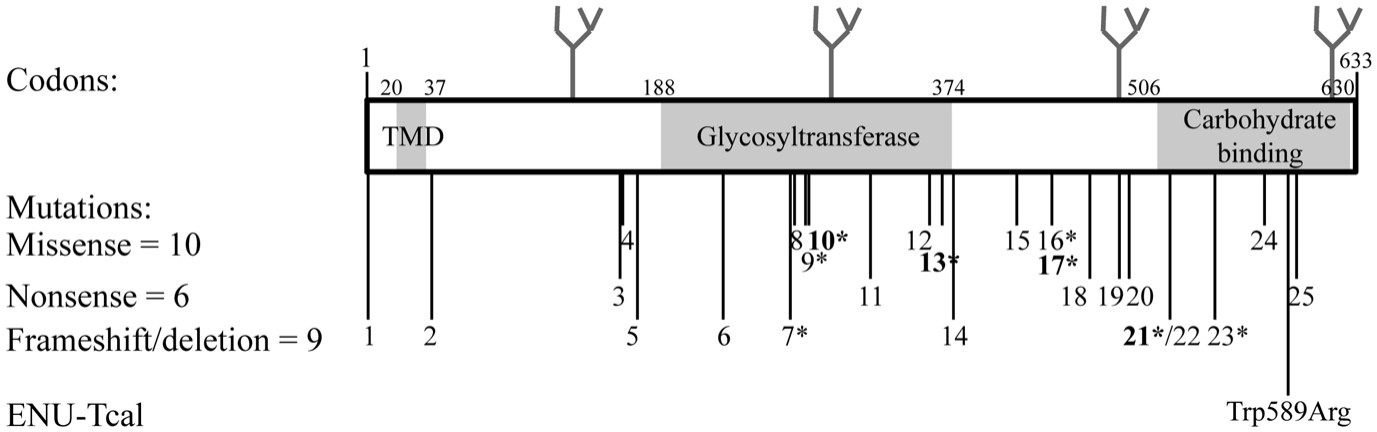
Schematic representation of GALNT3 structure. GALNT3 contains 3 domains which comprise a transmembrane domain (TMD) which is formed by residues 20 to 37; a glycosyltransferase domain which is formed by residues 188 to 374; and a carbohydrate-binding domain which is formed by residues 506 to 630, and contains two QXW repeats formed by residues 587–589 and 625–627, respectively. Human GALNT3 has four potential *N*-linked glycosylation sites (shown as branches) at amino acid residues 132, 297, 484 and 619, respectively [61]; whereas mouse Galnt3 has two potential *N*-linked glycosylation sites (not shown) at amino acid residues 297 and 484 (NetNGlyc 1.0). Twenty five *GALNT3* mutations (10 missense, 6 nonsense and 9 frameshift/deletion) have been reported in patients with familial tumoural calcinosis (FTC) and hyperostosis-hyperphosphataemia syndrome (HHS) (asterisked); and details of these 25 *GALNT3* mutations are provided in Table 1. Four *GALNT3* mutations (Glu281Gly, Leu366Arg, Arg438Cys and 464–508 deletion) have been reported in patients with FTC and HHS (bold and asterisked), thereby indicating that these 2 disorders are allelic variants [3], [6], [18], [19], [24]. The location of the ENU-induced mouse TCAL Trp589Arg mutation, which involved an evolutionary conserved Trp (W) residue (Fig. 3D), within the carbohydrate-binding domain, is shown relative to *GALNT3* mutations identified in man.

Tumoural Calcinosis (TC) is an autosomal recessive metabolic disorder which is characterized by progressive deposition of calcium phosphate crystals in peri-articular skin and soft tissues that often result in painful skin ulcerations, and secondary infection of skin and bone [1], [2]. Patients with TC may also have dental abnormalities, retinal angiomatoid streaks and vascular calcification [3]. The biochemical hallmark of TC is hyperphosphataemia which is caused by increased renal tubular reabsorption of phosphate [4]. Patients with TC may also have elevated or inappropriately normal plasma concentrations of 1,25-dihydroxyvitamin D, although circulating parathyroid hormone (PTH) and calcium concentrations are normal [2], and normal or elevated alkaline phosphatase activity [5]. TC and its familial (FTC) form may be caused by mutations of either the UDP-N-acetyl-alpha-D-galactosamine polypeptide N-acetyl galactosaminyl transferase 3 (*GALNT3*) [2], [6]–[10], fibroblast growth factor 23 (*FGF23*) [1], [10]–[12], or KLOTHO (*KL*) [13] genes. Thus, a total of at least 25 *GALNT3* homozygous mutations consisting of 10 missense [3], [14]–[19], 6 nonsense [6], [8], [9], [20], [21] and 9 frameshift/deletion [5]–[8], [16], [17], [22]–[25], (Fig. 1 and Table 1) have been reported in patients with FTC and the hyperostosis-hyperphosphataemia syndrome (HHS), which are allelic variants [2]; 3 homozygous missense FGF23 mutations (Ser71Gly, Met96Thr and Ser129Phe) have been reported in patients with TC [1], [11], [12]; and one homozygous missense KLOTHO mutation (His193Arg) [13] has also been reported in patients with TC. Moreover, GALNT3, FGF23 and KLOTHO form part of a phosphate regulating pathway [26]. For example, GALNT3 initiates a mucin type O-glycosylation and is known to selectively O-glycosylate a furin-like convertase recognition sequence in FGF23, which prevents proteolytic processing of FGF23 and allows secretion of intact FGF23 [27]. Indeed, patients with TC due to *GALNT3* and *FGF23* mutations have low circulating concentrations of intact FGF23 [16]. FGF23 inhibits the expression of the renal 25-hydroxyvitamin D-1α-hydroxylase (*Cyp27b1*), thereby decreasing serum concentrations of 1,25-dihydroxyvitamin D which results in a reduction of intestinal phosphate absorption. In addition, FGF23 which requires the co-receptor KLOTHO for bioactivity, downregulates the renal type II sodium-phosphate co-transporters (NPT2a (Slc34a1) and NPT2c (Slc34a3)) [28], [29], thereby decreasing renal tubular reabsorption of phosphate and leading to phosphaturia. *In vivo* studies of phosphate homeostasis have been greatly advanced by the availability of *Fgf23* [30] and *Kl* [31] null mice. The availability of a *Galnt3* null mouse, which was not available at the commencement of our study, would further facilitate investigations of phosphate homeostasis, and we therefore pursued studies to establish such a mouse model by assessing the progeny of mice treated with the chemical mutagen *N*-ethyl-*N*-nitrosourea (ENU) [32] for the occurrence of soft tissue (ectopic) calcification in association with hyperphosphataemia.

**Table 1:** *GALNT3* mutations identified in familial tumoural calcinosis (FTC) and hyperostosis-hyperphosphataemia syndrome (HHS) patients.

No.^a^	Mutation^b^	Codon	Predicted effect^c^	Clinical disorder^d^	References
	Exon 1				
1	c.T2A	1	p.Met1, loss of start codon	HHS	[17]
2	c.42_57 del	14	p.Arg14Ser fsX26	FTC	[22]
3	c.C484T	162	p.Arg162X	FTC	[6], [7]
4	c.G485A	162	p.Arg162Gln	FTC	[16]
	Exon 2				
5	c.516–2A>T → skip	173	p.Cys173Leu fs176 X	FTC	[5], [7]
6	c.677delC	226	p.Ala226Val fs228X	FTC	[16]
	Exon 3				
7	c.803–804insC	269	p.Thr269Asn fs281X	HSS	[23]
8	c.C815A	272	p.Thr272Lys	FTC	[14]
	Exon 4				
9	c.G839A	280	p.Cys280Tyr	HHS	[17]
10	c.A842G	281	p.Glu281Gly	FTC/HHS	[3]
11	c.T966G	322	p.Tyr322X	FTC	[20]
	Exon 5				
12	c.C1076A	359	p.Thr359Lys	FTC	[14]
13	c.T1097G	366	p.Leu366Arg	FTC/HHS	[3]
14	c.1102–1103 insT	368	p.Ser368Phe fs371X	FTC	[8]
	Exon 6				
15	c.T1245A	415	p.His415Gln	FTC	[18]
16	c.G1313A	438	p.Arg438His	HHS	[15]
17	c.C1312T	438	p.Arg438Cys	FTC/HHS	[18], [19]
18	c.A1387T	463	p.Arg463X	FTC	[21]
	Exon 7				
19	c.C1441T	481	p.Gln481X	FTC	[20]
20	c.G1460 A	487	p.Trp487X	FTC	[8]
21	c.1524+1G>A→ skip exon7	464	Del 44aa at codons 464–508	FTC, HHS, FTC/HHS	[6], [24], [25]
22	c.1524+5G>A→ skip exon7	464	Del 44aa at codons 464–508	FTC	[6]
	Exon 8				
23	c.1626+1G>A→ skip exon8	509	Del 34aa at codons 509–542	HHS	[23]
	Exon 9				
24	c.T1720G	574	p.Cys574Gly	FTC	[16]
25	c.C1774T	592	p.Gln592X	FTC	[9], [19]

a The locations of these mutations are illustrated in Figure 1.

b Nucleic acid change at base pair in the cDNA sequence (Genbank accession number NM_004482.3); del, deletion; skip, exon skipping; and ins, insertion.

c aa, amino acid; del, deletion; fs, frameshift. Mutations 2, 4, 5, 6, 14, 15, 16, 17, 18, 20, 21, 24 and 25 were identified as homozygotes. Of these, mutations 5, 14, 17, 21 and 25 were also identified in other patients as compound heterozygotes. Compound heterozygous mutations were identified in the following combinations: 1+9, 3+5, 3+21, 7+23, 8+12, 10+14, 11+19, and 17+25.

d FTC, familial tumoural calcinosis; HHS, hyperostosis-hyperphosphataemia syndrome.

## Results

### Phenotypic Identification of Tumoural Calcinosis (TCAL) Mice

**Figure 2:**
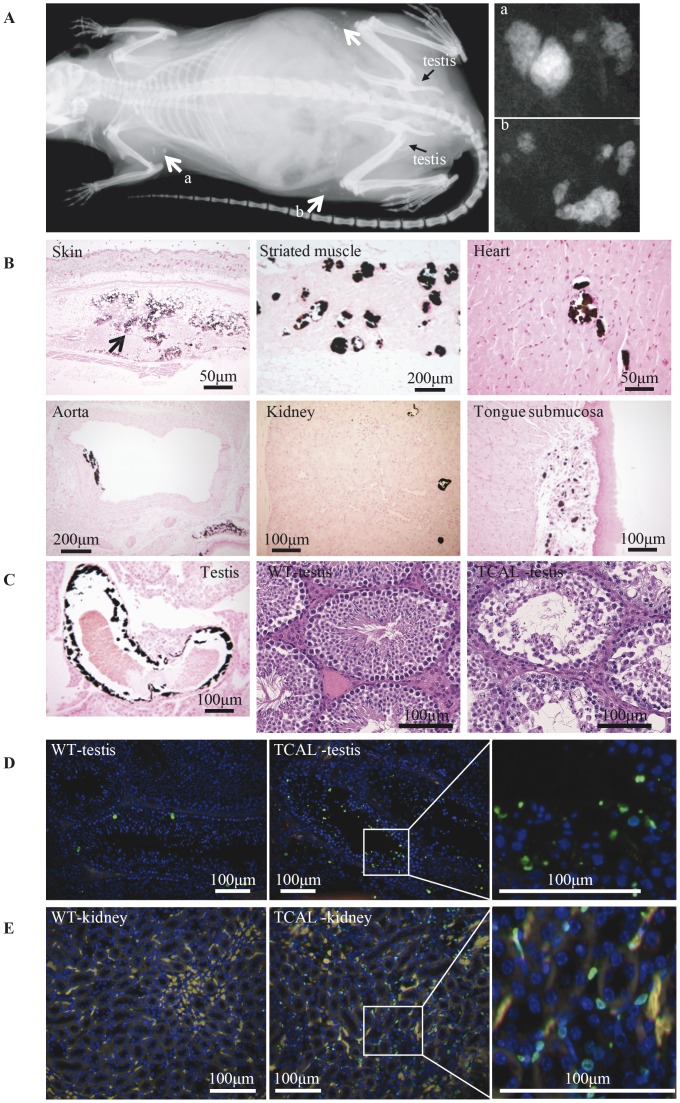
Phenotypic features of TCAL mice. (**A**) Radiography of an adult (12 week old) TCAL male mouse revealed widespread subcutaneous soft tissue opacities (white arrows); two magnified areas (a and b) are shown to illustrate detailed views. (**B**) Von Kossa stained sections from TCAL male mouse (36 week old) showing ectopic calcification in skin (arrow indicates mineralised panniculus fat), cutaneous striated muscle, heart, aorta, kidney and tongue submucosa. (**C**) Analysis of testes. Calcification was observed in the testicular arteries (left panel, von Kossa staining), and H&E sections (middle and right panels) of wild type (WT) and TCAL testis revealed disorganised seminiferous tubules and loss of spermatozoa in TCAL mice. (**D** and **E**) TUNEL staining of WT and TCAL testis and kidney sections, respectively, revealed increased apoptosis (green staining) in the tissues of TCAL mice.

Plasma biochemical analysis, at 12 weeks of age, of 14 G3 progeny (10 males and 4 females) derived from matings between parents and their offspring to yield autosomal recessive phenotypes revealed three mice (2males and 1 female) to have plasma phosphate concentrations of 3.53 mmol/l, 3.10 mmol/l and 2.87 mmol/l, which represented values that were >+3 standard deviations (SD) above the mean plasma phosphate for matched wild-type G3 other unrelated cohort controls (mean ±SD = 1.90±0.28 mmol/l, n = 80 (28 males and 52 females). Radiography revealed these 3 mice to have widespread soft tissue opacities (Fig. 2A). Thus, these mutant mice which had ectopic calcification in association with hyperphosphataemia, displayed phenotypic traits reminiscent of TC and the phenotype was designated TCAL and the locus, *Tcal*. Dental and retinal abnormalities were not identified in these TCAL mice. Breeding of affected TCAL males (2 mice from the original G3 progeny and 2 newly bred affected G3 mice, aged 10–16 weeks) with 8 different wild-type C3H females failed to yield any pregnancies, thereby suggesting that the TCAL males were infertile. However, TCAL female mice were fertile, and interbreeding of their progeny confirmed that TCAL was inherited as an autosomal recessive trait.

### TCAL Mice have Ectopic Calcification, Testicular Abnormalities and Increased Apoptosis

Von Kossa staining of tissues from the 3 TCAL affected G3 mice, described above, and 3 unaffected littermates (2 males and 1 female) revealed ectopic calcifications in subcutaneous tissues, cutaneous striated muscle, heart, aorta, kidney, tongue (Fig. 2B) and testicular artery (Fig. 2C) only in those sections from TCAL mice. In addition, haematoxylin and eosin (H&E) staining of TCAL mouse testes revealed disorganisation of the seminiferous tubules with a marked reduction of Sertoli cells and spermatozoa (Fig. 2C), consistent with a significant loss of germ cells and the observed infertility of these male TCAL mice. Terminal deoxynucleotidyl transferase dUTP nick end labeling (TUNEL) staining of testicular sections, revealed the TCAL male mice to have increased apoptosis in the lumen and periphery of seminiferous tubules, which likely involved spermatozoa, and Sertoli cells or spermatocytes (Fig. 2D), respectively. In addition, TUNEL staining of kidney sections revealed that TCAL male mice had increased apoptosis involving the interstitial cells in the renal medulla (Fig. 2E).

### Mapping of the *Tcal* locus to Chromosome 2C1.3-C2 and Identification of a *Galnt3* Missense Mutation

**Figure 3:**
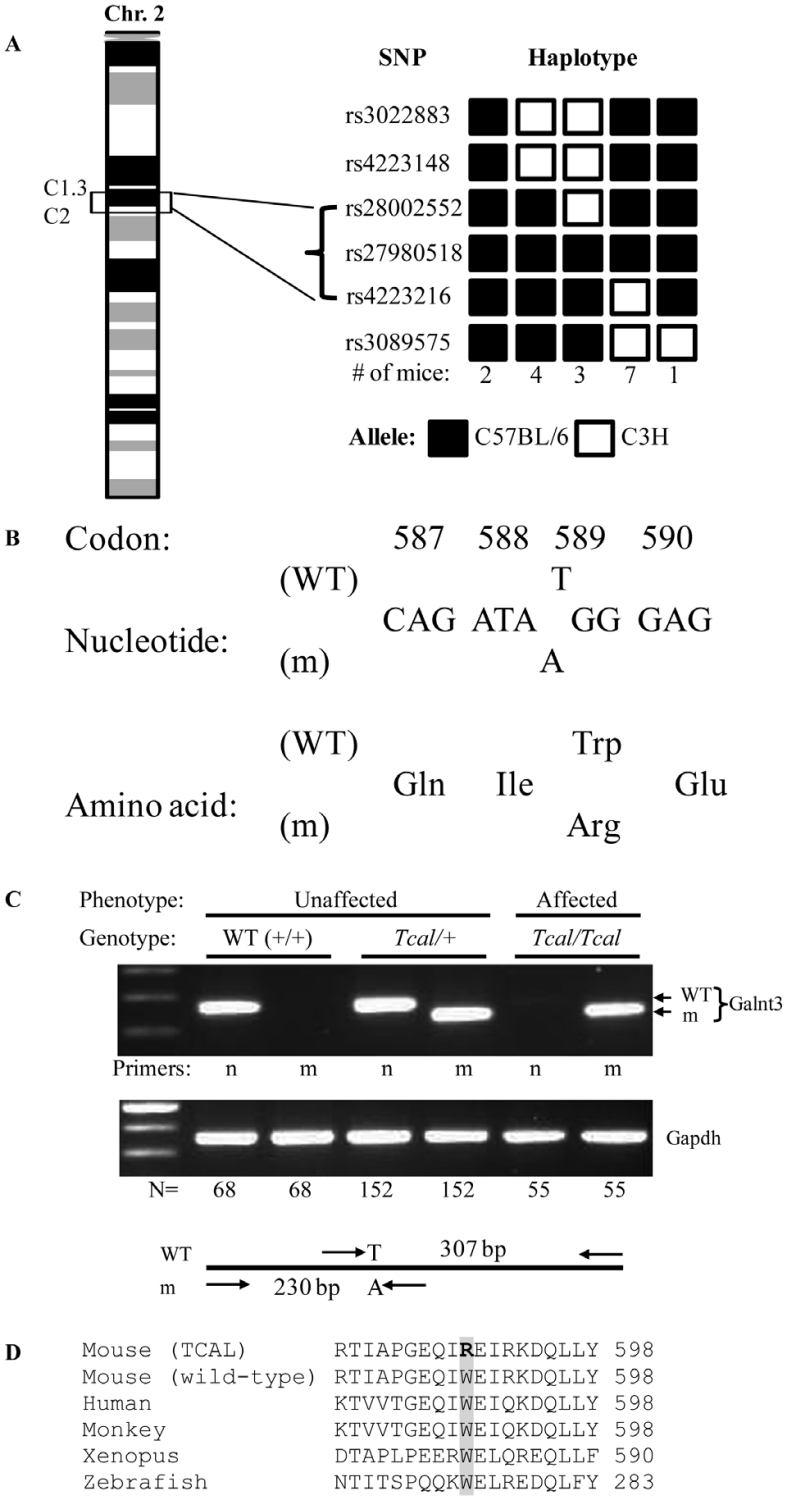
Mapping of *Tcal* locus and identification of *Galnt3* mutation. (**A**)The *Tcal* locus, which originated in a C57BL/6 ENU-mutagenised male and is hence inherited with the C57BL/6 alleles, was mapped to a 8.47 Mb region flanked by the SNPs rs28002552 and rs4223216 on chromosome 2C1.3–C2. This region contained 95 genes which included the *Galnt3* gene. (**B**) DNA sequence analysis of *Galnt3* identified a T to A transversion in codon 589, such that the wild type (WT) sequence, TGG which encodes an evolutionarily conserved tryptophan (Trp) residue was altered to the mutant (m) sequence, AGG which encodes an arginine (Arg) residue. (**C**) Amplification refractory mutation system (ARMS) PCR was used to confirm the presence of the mutation by designing primers (n, normal (WT) and m, mutant) that yielded 307 bp WT and 230 bp mutant PCR products, respectively. PCR amplification of *Gapdh* was used as a control for the presence of DNA. N = numbers of mice with each genotype. (D) Protein sequence alignment (CLUSTALW) of Galnt3 from 5 species revealed that the Trp (W) residue is evolutionarily conserved in the Galnt3 orthologues of mouse, human, monkey, xenopus and zebrafish.

Genome-wide mapping using DNA samples from 17 affected TCAL G5 mice (10 males and 7 females) and 91 SNP sets localised the *Tcal* locus to a 8.47 Mb region (between 62.64 and 71.11 Mb) flanked by rs28002552 and rs4223216 on chromosome 2C1.3–C2 (Fig. 3A). This interval contained 95 genes, which included *Galnt3* [2]. DNA sequence analysis of the *Galnt3* gene revealed a T to A transversion at codon 589 that resulted in a missense mutation Trp589Arg (Fig. 3B). The mutation was confirmed using the amplification refractory mutation system (ARMS) PCR method [33]. Thus, PCR using wild-type (WT)-specific primers yielded a 307 bp product only in DNA from unaffected mice (WT or heterozygous (*Tcal*/+)), whereas mutant-specific primers yielded a 230 bp product only in DNA from TCAL affected mice (*Tcal/Tcal*) or unaffected heterozygotes (*Tcal*/+) (Fig. 3C). The Trp589Arg mutation was found to involve an evolutionary conserved Trp (W) residue (Fig. 3D) that is part of the first of two QXW repeats within the carbohydrate-binding domain of GALNT3 (Fig. 1).

### *In vitro* and *in vivo* Functional Characterization of Mutant Galnt3

**Figure 4:**
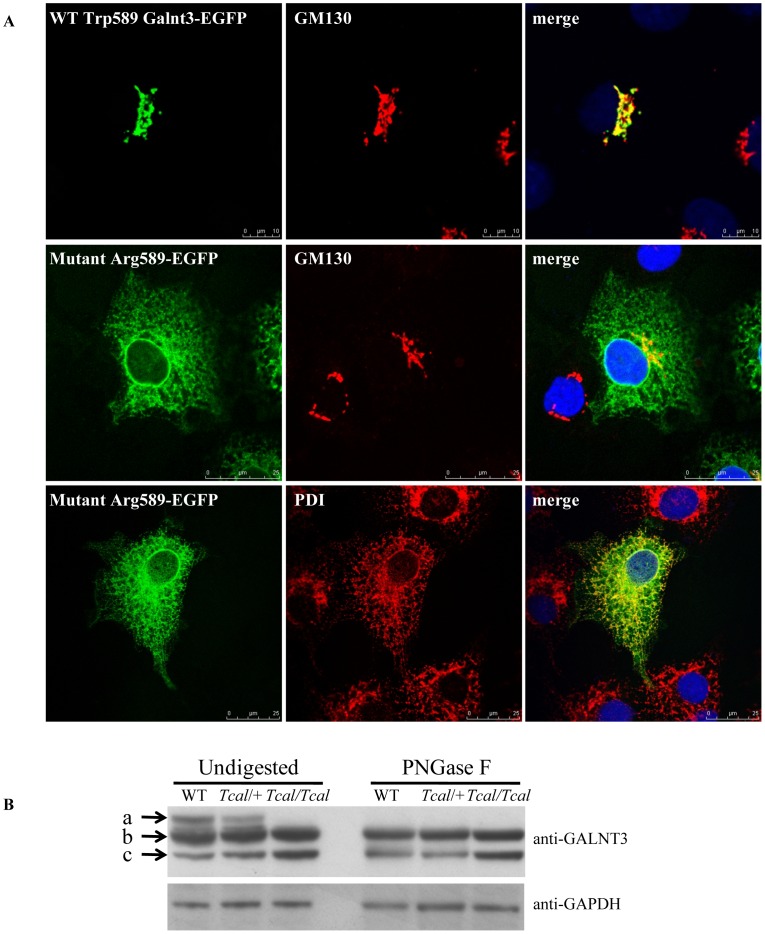
Mislocalization and defective glycosylation of mutant Galnt3. (**A**) COS-7 cells were transiently transfected with either EGFP-wild-type, WT (Trp589), or EGFP-mutant (Arg589) constructs, and counterstained with anti-GM130 antibody, which immunostains the Golgi apparatus (red), or anti-PDI antibody, which immunostains the ER (red). DAPI was used to stain the nucleus (blue). WT Galnt3 co-localizes with GM130, but not PDI (data not shown), thereby revealing that it is targeted to the Golgi apparatus. However, the mutant Galnt3 co-localizes with PDI and is predominantly found in the ER. (**B**) Western blot analysis of kidney homogenates using anti-GALNT3 antibody, revealed that protein lysates from WT littermates, and *Tcal*/+ mice had three immunoreactive products (a, b and c) whereas those from *Tcal/Tcal* mice had only two products (b and c). PNGase F treatment resulted in loss of the largest Galnt3 product (band a) observed in the lysates from WT littermates, and *Tcal*/+ mice, indicating that these were glycosylated products.

To investigate the functional consequences of the Trp589Arg Galnt3 mutation *in vitro*, WT and mutant enhanced green fluorescent protein (EGFP)-tagged *Galnt3* cDNA constructs were transfected in COS-7 cells and their sub-cellular localization assessed by immunofluorescence and confocal microscopy. WT Galnt3-EGFP, which co-localized with the Golgi marker, GM130 (Fig. 4A), was found to be expressed in the Golgi apparatus, whereas the expression pattern of the Arg589 mutant Galnt3 showed predominant co-localization with the endoplasmic reticulum (ER) marker, protein disulphide isomerase (PDI) (Fig. 4A), thereby suggesting impaired trafficking and ER retention of the mutant protein. Further investigation of the *in vivo* functional consequences of this Arg589 Galnt3 mutation revealed an effect on glycosylation (Fig. 1 and 4B). Thus, incubation of kidney homogenates from WT littermates, *Tcal*/+ and *Tcal/Tcal* mice in the presence or absence of the deglycosylating enzyme PNGase F and examination of the products by Western blot analysis using an anti-GALNT3 antibody, revealed that the kidney homogenates from both WT littermates and *Tcal*/+ G3 mice had three processed Galnt3 products, one of which was undetectable upon PNGase F digestion, thereby indicating that this was a glycosylated product; in contrast, the kidney homogenate from *Tcal/Tcal* mice lacked the glycosylated form of Galnt3, thereby indicating a defective glycosylation of the mutant protein (Fig. 4B).

### Plasma Biochemistry Analysis

**Figure 5:**
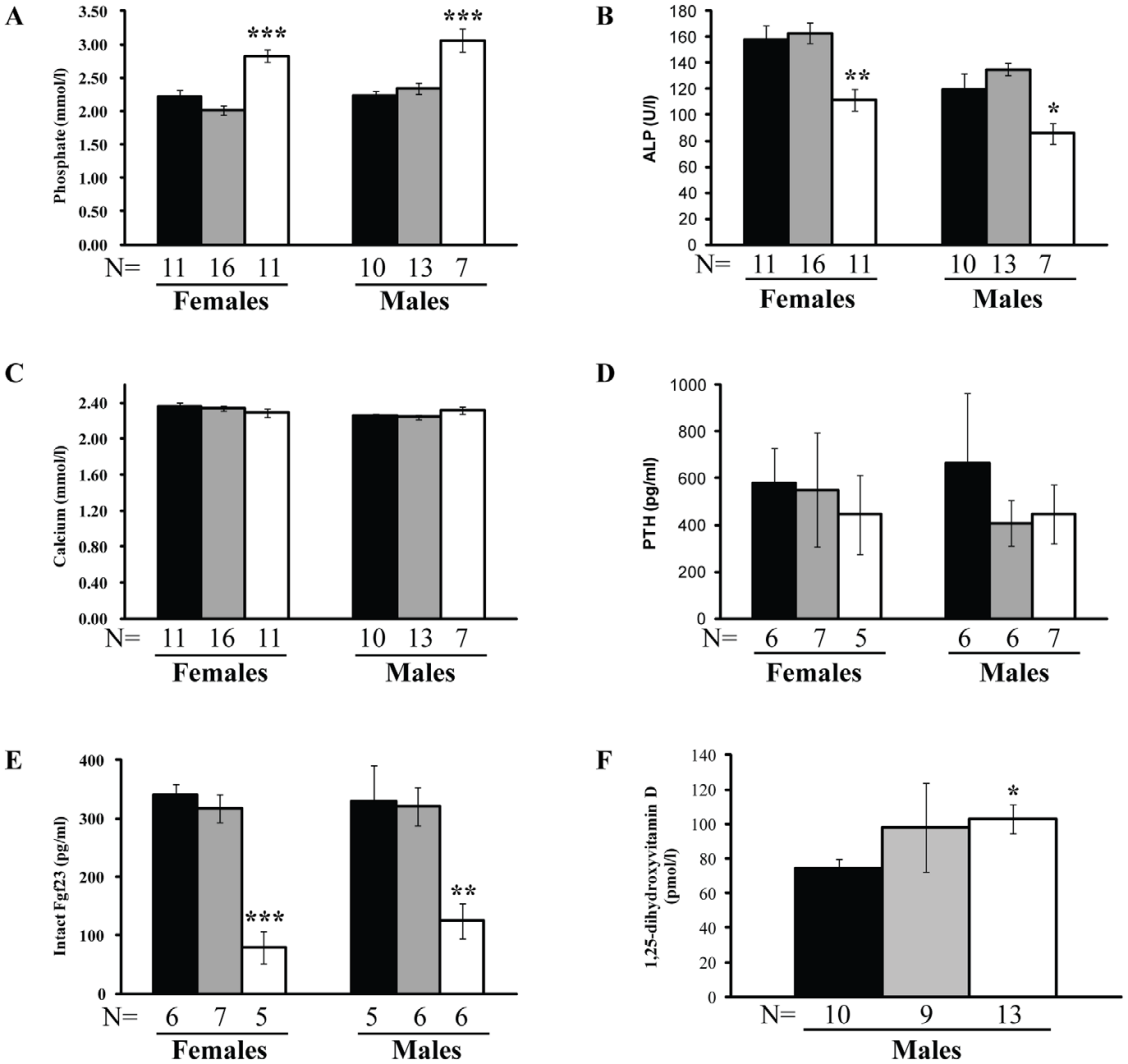
Plasma biochemical analyses. Plasma from WT littermates (+/+) (black), *Tcal*/+ (grey) and *Tcal/Tcal* (white) adult mice, aged 10 weeks was obtained and used to measure plasma concentrations of (**A**) phosphate, (**B**) alkaline phosphatase (ALP) activity, (**C**) calcium (adjusted for albumin concentrations), (**D**) PTH, (**E**) intact Fgf23, and (**F**) 1,25-dihydroxyvitamin D. *Tcal/Tcal* mice but not *Tcal*/+ mice, had: hyperphosphataemia, reduced alkaline phosphatase activity, reduced plasma concentrations of intact Fgf23, and elevated circulating concentrations of 1,25-dihydroxyvitamin D. The plasma concentrations of calcium and PTH were not significantly different. Sufficient volumes of plasma samples from female mice were not available for an analysis of 1,25-dihydroxyvitamin D concentrations; however, analysis of pooled samples from 2 WT female littermates, 3 *Tcal*/+ females, and 3 *Tcal/Tcal* females revealed that the plasma 1,25-dihydroxyvitamin D concentrations were 58.2 pmol/l, 55.7 pmol/l, and 71.7 pmol/l, respectively. These results indicate that the plasma 1,25-dihydroxyvitamin D concentrations in WT littermates and *Tcal*/+ females are similar, but are elevated in *Tcal/Tcal* females, and are consistent with the observations in male mice. The data are represented as mean ± SEM, and p-values are from unpaired Students t-test (*p<0.05, **p<0.01, ***p<0.001).

Plasma was collected from adult G3–G5 mice (n = 68) from over 10 weeks of age and these consisted of 21 WT (+/+) littermates (10 males and 11 females), 29 *Tcal*/+ mice (13 males and 16 females), and 18 *Tcal/Tcal* mice (7 males and 11 females). *Tcal/Tcal* mice, but not *Tcal*/+ mice had significantly elevated plasma phosphate concentrations (p<0.001) (Fig. 5A) and reduced plasma alkaline phosphatase activity (p<0.05) (Fig. 5B) when compared to WT littermates. The plasma calcium (Fig. 5C) and PTH concentrations (Fig. 5D) were similar in the WT, *Tcal*/+ and *Tcal/Tcal* adult mice. *Tcal/Tcal* adult mice, but not *Tcal*/+ adult mice, had significantly reduced plasma concentrations of intact Fgf23 (p<0.01) (Fig. 5E), but elevated circulating concentrations of 1,25-dihydroxyvitamin D (p<0.05) (Figure 5F), when compared to WT littermates.

### Assessment of Bone Structure by Micro-computed Tomography

**Figure 6:**
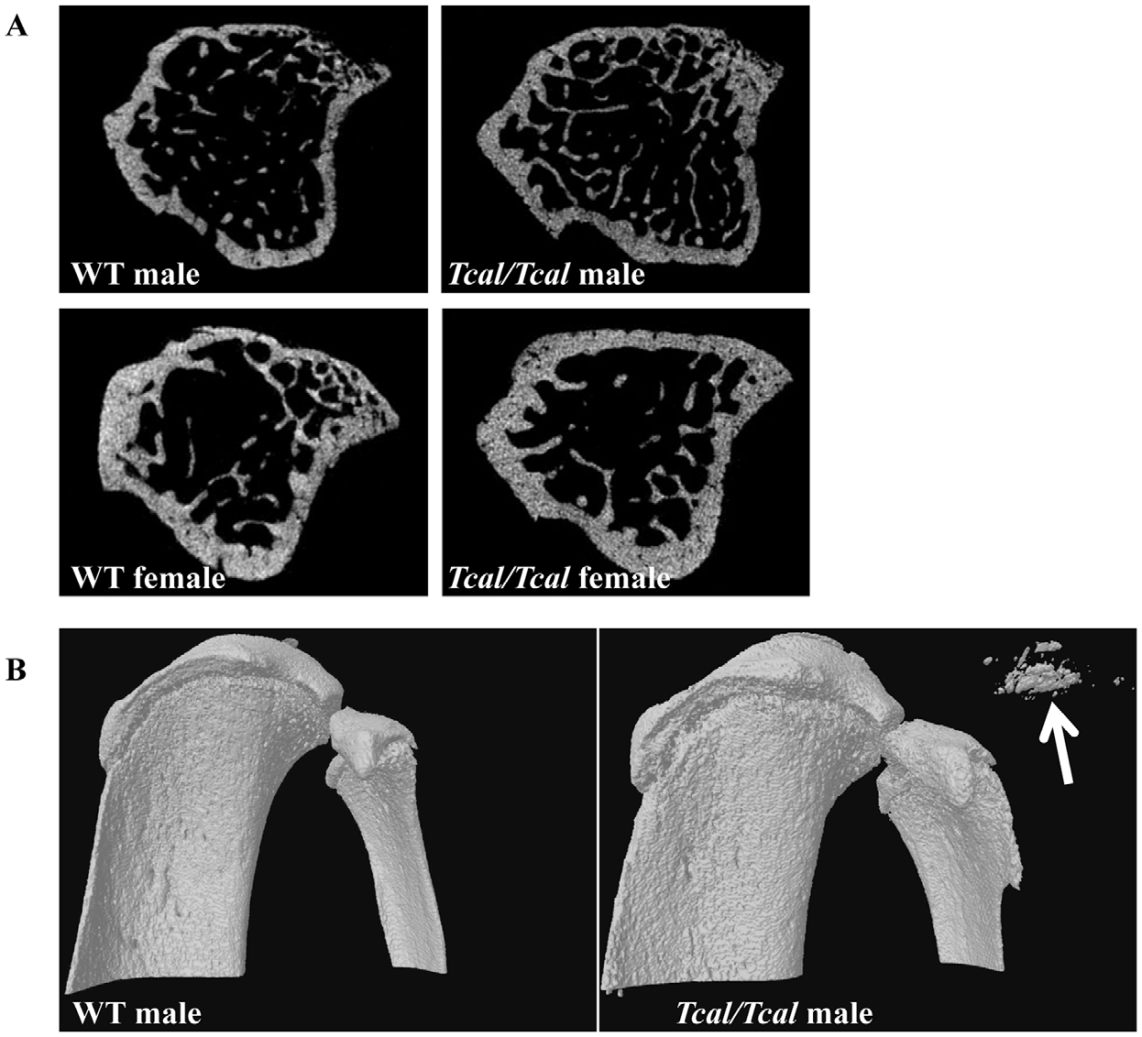
Micro-CT analysis. (**A**) Cross sections of proximal tibia from male and female mice revealed an increase in cortical bone volume in *Tcal/Tcal* mice (aged 12 weeks) when compared to WT littermates. (**B**) Three dimensional views of tibiae revealing ectopic calcification (arrowed) in a *Tcal/Tcal* male mouse (aged 12 weeks).

Dual-energy X-ray absorptiometry (DEXA) and microcomputed tomography (CT), was performed in 53 G3–G5 (31 males (8 WT littermates, 12 *Tcal*/+, 11 *Tcal/Tcal*) and 22 females (7 WT littermates, 10 *Tcal*/+ and 5 *Tcal/Tcal*)) and 18 G5 (9males (4 WT littermates and 5 *Tcal/Tcal*) and 9 females (4 WT littermates and *Tcal/Tcal*)) adult mice, respectively, that were >12 weeks of age. Areal bone mineral density (BMD), assessed using DEXA in >12 week old mice, has been previously reported [34] to be significantly elevated in *Tcal/Tcal* mice, but not *Tcal*/+ mice, when compared to WT littermates (mean BMD±SD: males (WT = 0.0580±0.0029 g/cm^2^, n = 8; *Tcal*/+ = 0.0610±0.0062 g/cm^2^, n = 12; *Tcal/Tcal* = 0.0669±0.0053 g/cm^2^, n = 11, p<0.001) and females (WT = 0.0565±0.0028 g/cm^2^, n = 7; *Tcal*/+ = 0.0572±0.0041 g/cm^2^, n = 10; *Tcal/Tcal* = 0.0633±0.0027 g/cm^2^, n = 5) p<0.001). We therefore further assessed bone structure by micro CT, in *Tcal/Tcal* mice and WT littermates (Fig. 6A), but not *Tcal*/+ mice, as their areal BMD was previously reported to be similar to that of WT littermates [34]. Micro-CT analysis of tibiae (Fig. 6A) revealed that cortical bone volume was significantly increased in male and female *Tcal/Tcal* mice. In addition, trabecular number was increased, and the structural model index decreased in male *Tcal/Tcal* mice (Table 2). Finally, cross-sectional analysis revealed the presence of calcinosis in soft tissues adjacent to the knee (Fig. 6B), elbow (not shown) and shoulder (not shown) in *Tcal/Tcal* mice but not WT littermates.

**Table 2:** Bone volume, assessed by micro-CT of tibiae, from 12 week old mice.

	Males		Females	
	WT (+/+) (n = 4)	*Tcal/Tcal* (n = 5)	WT (+/+) (n = 4)	*Tcal/Tcal* (n = 5)
BV/TV (%)	10.3±1.3	17.7±3.0	14.4±2.4	18.8±1.1
Tb. Th (mmx10^−2^)	4.2±0.05	4.0±0.1	5.1±0.2	5.4±0.2
Tb. N (mm^−1^)	2.4±0.3	4.4±0.7*	3.3±0.7	1.9±0.3
SMI	1.6±0.2	0.7±0.1**	1.5±0.3	1.1±0.3
C. BV (mm^3^)	0.98±0.03	1.16±0.04**	0.89±0.08	1.16±0.04*

WT (+/+), refers to wild-type littermates; BV/TV  =  trabecular bone volume (BV) fraction as a proportion of total volume (TV) of organ; Tb. Th  =  trabecular thickness, Tb. N  =  trabecular number; SMI  =  structural model index; and C.BV  =  cortical bone volume. Values represent means ± SEM; Unpaired student’s t-test: *p<0.05, **p<0.01.

### Effects of *Galnt3* Mutation on Gene Expression in Bone and Kidney

**Figure 7:**
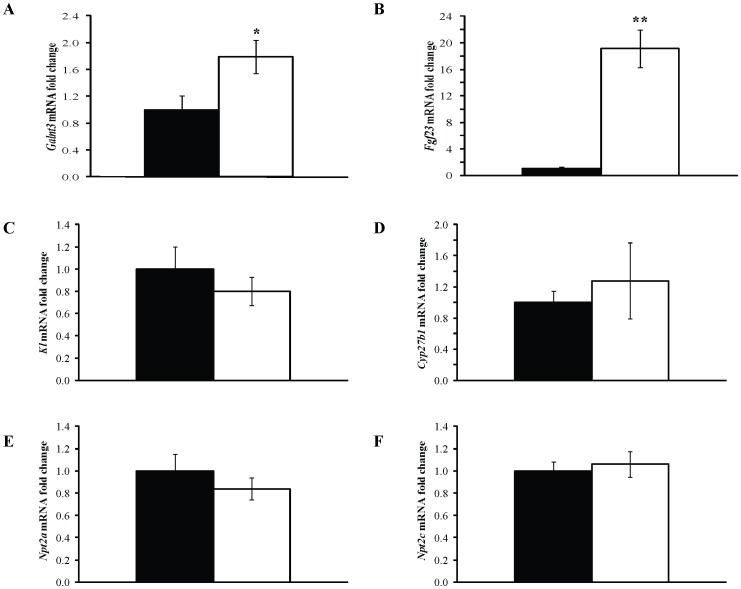
Analysis of gene expression in bone and kidneys. RNA from femora and kidneys was extracted from WT littermates (black) (2 males and 1 female) and *Tcal/Tcal* (white) (2 males and 1 female) adult mice, aged 18–20 weeks. Quantitative reverse transcriptase-PCR (qRT-PCR) was used to study the expression of: (**A**) *Galnt3* and (**B**) *Fgf23* in femora; and (**C**) *Kl*, (**D**) *Cyp27b1*, (**E**) *Slc34a1*, and (**F**) *Slc34a3* in kidneys. Samples were analysed in triplicate (n = 3 mice for each group i.e. total of 9 samples) and mRNA levels were normalized to *Gapdh* and expressed as fold change (mean ± SEM) compared to WT. The data from males and females were combined, as differences in plasma biochemical analysis between the genders had not been observed (Fig. 5). The expression of *Galnt3* and *Fgf23* was significantly increased in the bone of *Tcal/Tcal* mice when compared to that of WT littermates; however, the expression of the renal expressed genes *Kl*, *Cyp27b1*, *Slc34a1* and *Slc34a3* was not significantly different in the *Tcal/Tcal* mice compared to WT littermates. P-values are from unpaired Students t-test (*p<0.05, **p<0.01).

Femora and kidneys were obtained from 3 WT littermates (2 males and 1 female) and 3 *Tcal/Tcal* (2 males and 1 female) adult G5 mice that were >18 weeks of age. RNA was extracted and gene expression investigated by quantitative reverse transcriptase-PCR (qRT-PCR). *Tcal*/+ mice were not studied, as plasma biochemistry (Fig. 5) and areal BMD [34] analysis had not revealed any significant differences when compared to WT littermates. Data from *Tcal/Tcal* male and female mice were combined as analysis of plasma biochemistry (Fig. 5) and areal BMD [34] had revealed similar abnormalities when compared to WT littermates. The expression of *Galnt3* and *Fgf23* was studied in femora, and this revealed that *Tcal/Tcal* mice had significantly increased expressions of *Galnt3* (Fig. 7A) and *Fgf23* (Fig. 7B) by 1.8-fold and 19-fold, respectively, when compared to that in WT littermates. The higher *Fgf23* expression contrasts with the lower circulating concentrations of Fgf23, suggesting that there is a loss of negative feedback in the *Tcal/Tcal* mice (Fig. 5E). The effects of the reduced circulating concentrations of Fgf23 on the renal expression of *Klotho* (*Kl*) [26] (Fig. 7C), vitamin D 1-alpha hydroxylase (*Cyp27b1*) (Fig. 7D) and the renal sodium-phosphate co-transporters (*Npt2a*) [28] (Fig. 7E) and *Npt2c* [29] (Fig. 7F), were investigated; however, these were found to be similar in *Tcal/Tcal* mice and WT littermates. Thus, the observed plasma biochemical abnormalities in phosphate (Fig. 5A and 5B) and 1,25-dihydroxyvitamin D (Fig. 5F) homeostasis, could not be attributed to any possible effects of reduced plasma Fgf23 concentrations on renal expression of *Npt2a*, *Npt2c*, and *Kl;* or *Cyp27b1*, respectively.

## Discussion

Our study describes a mouse model (TCAL) with an ENU-induced *Galnt3* mutation that has similarities to familial tumoural calcinosis (FTC) in man (Table 3). Thus, TCAL mice had hyperphosphataemia in association with ectopic calcification. Moreover, TCAL mice had increased circulating concentrations of 1,25-dihydroxyvitamin D, and decreased plasma intact Fgf23 concentrations. TCAL was inherited as an autosomal recessive disorder, consistent with the inheritance of FTC, and due to a missense Trp589Arg Galnt3 mutation (Fig. 3B and 3C) that was induced by ENU, which is known to induce multiple mutations simultaneously [32]. However, the likelihood that another genetic defect within the 8.47 Mb region that was established to be the location of the *Tcal* locus (Fig. 3A), could be the underlying cause of TCAL is <0.01, based on the following reasoning. The nominal ENU induced base pair mutation rate for potentially functional mutations has been estimated to be 1 in 1.82 Mb of coding DNA in the F1 founder animals [35] and given that <2.5% of the mouse genome is coding, it has been calculated that the probability of two functional mutations arising within a 5 Mb genomic region is <0.002 [36]; thus, the likelihood of the Galnt3 Trp589Arg and another functional mutation arising within the 8.47 Mb containing the *Tcal* locus is <0.004. This indicates that the Galnt3 Trp589Arg mutation, which was shown also to result in ER retention of the mutant protein (Fig. 4A), as well as defective glycosylation (Fig. 4B), is highly likely to be the sole genetic defect causing TCAL. Although the Trp589Arg missense Galnt3 mutation associated with TCAL in the mouse has not been identified in patients with FTC, it is important to note that Trp589 is conserved in both species and that the Trp589Arg mutation is representative of 40% of GALNT3 abnormalities which are also missense mutations in patients with FTC and HHS [3], [14]–[19].

**Table 3:** Comparison of *Galnt3*-deficient and TCAL mouse models with FTC and HHS in man.

Main characteristics	*Galnt3* ^−/−^ mice^a^	*Tcal/Tcal* mice^b^	FTC in man^c^	HHS in man^d^
Derivation of mutation	KO	ENU	SN	SN
Ectopic calcification	−	+	+	−
Growth retardation in males	+	−	−	−
Infertility in males	+	+	?	?
Hyperphosphataemia	+	+	+	+
Plasma alkaline phosphatase activity	↓	↓	N/↑	N
Plasma calcium concentration	↑	N	N	N
Circulating PTH	↓	N	N	N
Circulating1,25-dihydroxyvitamin D	N	↑	N/↑	N/↑
Reduced circulating intact Fgf23	+	+	+	+
Increased areal BMD	+, in males only	+, males and females*	?	?
Apoptosis in testis and kidney	?	+	?	?
*Fgf23* expression in bone	↑	↑	?	?
*Galnt3* expression in bone	?	↑	?	?
*NPT2a* expression in kidney	↑	N	?	?
*Kl* expression in kidney	↑	N	?	?

KO, knockout model; ENU, *N*-ethyl-*N*-nitrosourea induced model; SN, spontaneous naturally occurring.

+, present; −, absent; N, normal; ↓, reduced; ↑, increased; ?, not reported.

* Previously reported (reference [34]).

a previously reported (reference [31]);

b this study;

c previously reported (references [3], [5]–[9], [14], [16], [18]–[22], [24]);

d previously reported (references [3], [15], [17], [19], [23]–[25]).

During the course of our study, a *Galnt3*-deficient mouse was reported [31], and this mouse model and TCAL had some phenotypic features in common (Table 3). Thus, TCAL and *Galnt3*-deficient mice are characterized by the presence of hyperphosphataemia, decreased plasma alkaline phosphatase activity, reduced circulating intact Fgf23, increased Fgf23 gene expression in bone, increased whole body BMD in male mice, and male infertility due to loss of spermatozoa in seminiferous tubules. However, there are also important differences between TCAL and the *Galnt3*-deficient mice (Table 3) and these include: an absence of growth retardation in TCAL mice; elevated plasma 1,25-dihydroxyvitamin D concentrations in TCAL mice (Fig. 5F); normal plasma concentrations of calcium and PTH in TCAL mice; increased areal BMD in female TCAL mice; and ectopic calcification (Figs. 2A & 2B) in TCAL mice, which is a hallmark of FTC in man [4], but was notably absent in *Galnt3*-deficient adult mice, even when aged to 1 year [31]. The basis of these differences between TCAL and *Galnt3*-deficient mice remains to be elucidated. A possible explanation may involve strain-specific differences as the TCAL mice were on a mixed C57BL/6J and C3H background, whilst the *Galnt3*-deficient mice were on a C57BL/6J and 129SvEv background [31]. In addition, ENU-induced mouse models have been reported to differ in phenotypic features when compared to the corresponding null mice, generated using targeted gene ablation strategies [32]. For example, mice deficient for the fat mass and obesity associated (*FTO*) gene (*FTO* ^−*/−*^) have been reported to have phenotypic differences when compared to mice that were homozygous for the ENU hypomorphic mutant *FTO^I367F^*. Thus, *FTO* ^−*/−*^ and *FTO^I367F^* mice both have reduction in adiposity and weight, but only *FTO* ^−*/−*^ mice show perinatal lethality, and age-related reduction in size and length [37]. Another possibility that may contribute to these differences in severity of the phenotype may be related to the functions of other GALNTs, e.g. Galnt6 that can partially compensate for the loss of Galnt3 [31]. However, it is also important to note that there is significant variability in the clinical manifestations amongst FTC patients (Table 3). For example, FTC, in man, has a variable age of onset with variation in the severity of calcified lesions, such that some patients suffer from large extra-skeletal lesions that require surgery [4], [38], whilst others have mild disease that may be asymptomatic [14]. In addition, *GALNT3* mutations in man, may result in the hyperostosis-hyperphosphatemia syndrome, (HHS) [15], [17], [23], [24], in which cortical hyperostosis is a notable feature. However, the same *GALNT3* mutation may be associated with FTC and HHS in members of the same family or in unrelated families [6], [18], [19], [24]. Indeed, FTC and HHS are considered to be allelic variants and the situation between TCAL and *Galnt3*-deficient mice may be analogous. Thus, TCAL mice had ectopic calcifications (Fig. 2) and thickening of cortical bone (Table 2), consistent with FTC and HHS, whilst *Galnt3*-deficient mice did not have soft tissue calcification, but only had thickening of cortical bone, consistent with isolated HHS.

The three most notable differences between human FTC and mouse TCAL are the findings of decreased plasma alkaline phosphatase activity, and male infertility in TCAL mice which are not found in man, and the occurrence of smaller tumoural calcinosis lesions in TCAL mice. Interestingly, the *Galnt3*-deficient mouse also was reported to have these differences, and the basis of these inter-species phenotypic differences remains to be elucidated. The observation of decreased plasma alkaline phosphatase activity has in the *Galnt3*-deficient mice been attributed to be associated with the reported increased bone mineralization in these mutant mice [31]. Given the reported increased BMD [34] (Table 3) in the *Tcal/Tcal* mice, it would seem probable that the decreased plasma alkaline phosphatase activity in the *Tcal/Tcal* mice is also a reflection of increased bone mineralization. *Tcal/Tcal* and *Galnt3*-deficient male mice had infertility, and it is important to note that recent studies indicate that this is not due to the hyperphosphataemia, as normalizing the serum phosphate concentrations in *Galnt3*-deficient mice, by use of a low phosphate diet failed to correct the infertility [39]. Infertility in males with FTC or HHS is not a notable feature. However, one boy with FTC, has been reported to have testicular microlithiasis which was associated with oligoazoospermia, and histology revealed that the calcifications were localized to the lumen of the seminiferous tubules and the interstitium [21]. The FTC in this boy and his family did not co-segregate with an autoimmune disorder, which resulted in arthralgia, vasculitis and chronic immune thrombocytopenic purpura, thereby indicating that the oligoazoospermia was not due to the autoimmunity [21]. GALNT3 is highly expressed in the testis, and its loss may cause deposition of calcium in the testis; indeed, it has been suggested that testicular calcification may be an underestimated feature of FTC [21]. The differences in the sizes of the calcinosis lesions between human FTC and the *Tcal/Tcal* and *Galnt3*-deficient mouse models, may in part be attributed to the observed variability of FTC lesions in man [4], [14], [38]. However, they may also be related to dietary phosphate intake. For example, *Galnt3*-deficient mice when placed on diets containing either 0.1% (low), 0.3% (low normal), 0.6% (normal) or 1.65% (high) phosphate developed a significant increase in serum calcium concentrations when on the high-phosphate diet [39], although *Galnt3*-deficient mice, aged to 1 year, have not been observed to develop ectopic calcification when on a 0.93% phosphate diet [31]. It has been postulated that the hypercalcaemia induced by the 1.65% (high) phosphate diet may likely contribute to the overall increase in calcium-phosphate products and subsequently ectopic calcifications. Thus, it seems possible that the variability in the size of the tumoural calcinosis lesions in man, may be related to dietary phosphate, with high intake being associated with the larger lesions. Another possibility that may contribute to the variability in the size of the tumoural calcinosis lesions, may involve a response to injury. For example, it has been suggested that early calcinosis lesions are triggered by injury and bleeding, with subsequent aggregation of foamy histiocytes, which become transformed into cystic cavities lined by osteoclast-like giant cells, and surrounded by monocytes and iron-loaded macrophages [40], [41]. Studies investigating the responses to injury and the underlying inflammatory and immune mechanisms in the *Tcal/Tcal* mice, which have calcinosis lesions, and in the *Galnt3*-deficient mice, which do not have calcinosis lesions (Table 3), may help to elucidate the basis of these differences.

GALNT3 belongs to a large family of Golgi-resident glycosyltransferases that initiate mucin-type O-glycosylation, one of the most abundant forms of protein glycosylation found in eukaryotic cells [42], [43]. Structurally, GALNT3 consists of an N-terminal transmembrane domain, a central catalytic (glycosyltransferase) domain and a C-terminal ricin (carbohydrate binding) domain (Fig. 1). FTC and HHS mutations are distributed throughout the *GALNT3* gene, with no evidence for clustering. The Trp589Arg missense mutation identified in TCAL mice is situated in the carbohydrate binding domain (Fig. 1) which is characterized by the presence of QXW (glutamine-any amino acid-tryptophan) repeats [44], and two of these which are present in both human and mouse GALNT3 orthologues. The Trp589Arg mutation in TCAL mice alters the tryptophan residue in the first repeat. Each QXW repeat forms an omega loop and it has been suggested that these could be important for post-translational protein folding and stabilization, and for carbohydrate binding [44]. Indeed, our *in vitro* and *in vivo* studies of the Galnt3 Trp589Arg mutant, which alters the tryptophan in the first QXW repeat, demonstrated such roles for the QXW repeats by showing impaired trafficking of the mutant protein, with its retention in the endoplasmic reticulum (Fig. 4A) and defective glycosylation of the mutant Galnt3 protein in kidney lysates from *Tcal/Tcal* mice, respectively (Fig. 4B).

Studies of null mouse models of *FGF23* [30], *vitamin D-1-alpha hydroxylase* [45], *klotho* [46] and *NPT2a* [28] have established that FGF23 reduces serum phosphate levels by suppressing phosphate reabsorption in proximal kidney tubules [47], thereby playing a key role as a regulator of phosphate metabolism. FGF23 is O-glycosylated by GALNT3 to protect it from proteolytic cleavage [10], [48], and the underlying molecular mechanism causing FTC and HHS in patients with GALNT3 mutations involves defective glycosylation of FGF23 resulting in enhanced cleavage and inactivation of FGF23 [47]. Our results, which reveal a reduction in circulating concentrations of intact full-length Fgf23 in *Tcal/Tcal* mice, indicate that the Trp589Arg Galnt3 mutation is an inactivating mutation whose loss-of-function releases the inhibition on 1,25-dihydroxyvitamin D synthesis [49], as observed by increased plasma concentrations of 1,25-dihydroxyvitamin D. Furthermore, our *in vivo* results which show a 1.8 fold increase in bone expression of *Galnt3* in *Tcal/Tcal* mice in response to chronic hyperphosphataemia are in agreement with *in vitro* studies which showed that *GALNT3* gene expression can be induced by administration of extracellular phosphate to cultured human fibroblasts [50]. Moreover, our analysis, which revealed extensive apoptosis in testis (Fig. 2D) and kidney (Fig. 2E) in association with the prevailing hyperphosphataemia in *Tcal/Tcal* mice, is in agreement with *in vitro* studies that have reported that high levels of extracellular phosphate are a potent inducer of oxidative stress and apoptosis in cultured human endothelial cells [51] and osteoblast-like cells from human bone explants [52].

In summary, our study has identified a mouse model for autosomal recessive FTC due to an ENU-induced missense mutation (Trp589Arg) in Galnt3 and this will help to elucidate further the molecular mechanisms of FTC and provide a model for investigating novel treatments.

## Materials and Methods

### Ethics Statement

All animal studies were carried out using guidelines issued by the Medical Research Council in ‘Responsibility in the Use of Animals for Medical Research’ (July 1993) and Home Office Project License Number 30/2433. Experiments were approved by the Medical Research Council Harwell ethics committee.

### Generation of Mutant Mice

Male C57BL/6J mice were treated with ENU and mated with untreated C3H female mice [32]. The male progeny (G1) were subsequently mated with normal C3H females to generate G2 progeny. The female G2 progeny were backcrossed to the G1 fathers and the resulting G3 progeny [32] were screened at 12 weeks of age for recessive phenotypes. Mice were fed an expanded rat and mouse no. 3 breeding diet (Special Diets Services, Witham, UK) containing 1.15% calcium, 0.82% phosphate and 4088.65 units/kg vitamin D, and given water ad libitum. Wild-type littermates were used as controls, as these would have similar random assortments of segregating C57BL/6J and C3H alleles, to those of the mutant mice, thereby minimising any strain-specific influences.

### Plasma Biochemistry

Blood samples were collected from the lateral tail vein of mice [53] that had fasted for 4 hours. Plasma samples were analysed for total calcium, inorganic phosphate, alkaline phosphatase activity, urea, creatinine and albumin on a Beckman Coulter AU680 semi-automated clinical chemistry analyzer using the manufacturer’s instructions, parameter settings and reagents, as described [53]. Plasma calcium was adjusted for variations in albumin concentrations using the formula: ((albumin-mean albumin) ×0.02) + calcium), as described [54]. For analysis of PTH, FGF-23, and 1,25-dihydroxyvitamin D, blood samples were collected from the retro-orbital sinus after terminal anaesthesia, and plasma was separated by centrifugation at 3000 g for 5 min at 4°C. PTH was quantified using a two-site ELISA kit (Immunotopics, California, USA), intact FGF-23 was quantified using a two-site ELISA kit (Kainos Laboratoties, Tokyo, Japan), and 1,25-dihydroxy vitamin D was measured using an assay system (Immunodiagnostic Systems, Boldon, UK) involving purification by immunoextraction followed by quantification by enzyme immunoassay.

### Imaging by Radiography and Micro-CT Scanning

Anaesthetised mice were subjected to digital radiography at 26 kV for 3 seconds using a Faxitron MX-20 digital X-ray system (Faxitron X-ray Corporation, Lincolnshire, USA) [55]. Images were processed using the DicomWorks software (http://www.dicomworks.com/). For micro-CT scanning, formalin-fixed, undecalcified tibiae were used and analysed by a micro-CT scanner (model 1172a, Skyscan) at 50 kV and 200 µA utilizing a 0.5 aluminium filter and a detection pixel size of 17.4 µm^2^. The proximal tibia was scanned to measure trabecular bone [56], using a detection pixel size of 4.3 µm^2^, and images were scanned every 0.7° through a 180° rotation. Scanned images were reconstructed using Skyscan NRecon software and analyzed using the Skyscan CT analysis software (CT Analyser v1.8.1.4, Skycan). A volume of 1 mm^3^ of trabecular bone 0.2 mm from the growth plate was chosen. Trabecular bone volume as proportion of tissue volume (BV/TV, %), trabecular thickness (Tb.Th, mmx10^−2^), trabecular number (Tb. N, mm^−1^) and structure model index (SMI) were assessed in this region using the CT analysis software.

### Histology and Immunohistochemistry

Dissected tissues were fixed in 10% formalin, decalcified in formical-4™ (Decal Chemical Corporation) for 3 days before embedding in paraffin wax [55]. Sections (3–4 µm) were stained with haematoxylin and eosin (H&E), and von Kossa, for microscopy studies (Leica microscope model DM4000B, Leica Microsystems, Milton Keynes, UK) and images captured (QImaging camera model 10-RET-OEM-F-CLR-12, QImaging, Canada) [55]. TUNEL staining was performed on sections using the ApopTag Fluorescein In Situ Apoptosis Detection kit (Millipore) [57]. Stained sections were mounted in VECTASHIELD® mounting medium containing 4′,6-diamidino-2-phenylindole (DAPI) (Vector laboratories, Peterborough, UK) to detect nuclei [57].

### Mapping, DNA Sequence Analysis and Genotyping

Genomic DNA was extracted from tail or auricular biopsies, as described [53]. For genome-wide mapping, genomic DNA was amplified by PCR using a panel of 91 single nucleotide polymorphic (SNP) loci arranged in chromosome sets, and the products were analysed by pyrosequencing [55]. Individual exons of *Galnt3* were amplified from genomic DNA by PCR using gene-specific primers and Taq PCR Mastermix (Qiagen, Crawley, UK), and the PCR products sequenced using BigDye terminator reagents and ABI 3100 sequencer (Life Technologies, Carlsbad, USA). For genotyping, DNA was amplified by ARMS PCR using Taq PCR Mastermix (Qiagen, Crawley, UK) and specific primers for the wild-type (F: GACCATCGCCCCTGGAGAACAGACAT, R: AGAAGTTTTTCACCTACAGAAGCCAAGCGT) and mutant (F: CTTGTTTTATTTTGCAACTGGGCACAC, R: GAGCCAATCACCTTCCGAATCTCTCT) *Galnt3* sequences, and Glyceraldehyde 3-phosphate dehydrogenase (*Gapdh*) (F: CTCAGCTCCCCTGTTTCTTG, R: GGAAAGCTGAAGGTGACGG), and separated by agarose gel electrophoresis before image acquisition using a Gel Doc™ UV transilluminator (Bio-Rad, Hemel Hempstead, UK) [33].

### In vitro and in vivo Expression Studies of Wild-type and Mutant Galnt3

A full length mouse wild-type *Galnt3* cDNA was amplified from an IMAGE clone (IMAGE: 5342768) with Pfu Ultra II fusion (Agilent Technologies, Stockport, UK) using the forward primer (5′- CTCAAGCTTGACAGAATGGCTCACCTTAAGC -3′) and the reverse primer (5′- AGTGGATCCGA ATCATTTTGGCTAAAAATCCATT -3′), and the PCR product sub-cloned into pEGFP-N1 (Clontech, Saint-Germain-en-Laye, France) [58]. The *Galnt3* mutation was introduced using site-directed mutagenesis with the forward primer 5′-GGAGAACAGATA**A** GGGAGATTCGGA-3′ and its reverse complement, and sequence analysis of the constructs was undertaken using previously reported methods [58]. The wild-type and mutant Galnt3 constructs were transiently transfected into COS-7 cells using FuGENE 6 reagent (Roche, Welwyn Garden City, UK) and 1 µg of each construct as previously described [59] and expression visualized by immunofluorescence [60]. Briefly, transfected cells cultured on glass coverslips were fixed, permeabilized, blocked and incubated with either mouse anti-Golgi matrix protein (GM130) (BD Bioscience, Oxford, UK) or mouse anti-protein disulphide isomerase (PDI) (Enzo Life Science, Exeter, UK) diluted 1∶500. The secondary antibody was AlexaFluor 594 goat anti-mouse (Invitrogen, Paisley, UK) diluted 1∶500. Coverslips were mounted onto slides in VECTASHIELD® mounting medium with DAPI (Vector laboratories, Peterborough, UK) and visualized by confocal microscopy using a Leica TCS SP5 confocal system, attached to a DMI 6000 microscope. Western blot analysis was performed using equal amounts of proteins from kidney homogenates that were pre-incubated for 1 h at 37°C in the presence or absence of peptide: N-glycosidase F (PNGase F), mixed with LDS sample buffer (Invitrogen, Paisley, UK) before separation by sodium dodecyl sulfate-polyacrylamide gel electrophoresis (SDS-PAGE) and electroblotting onto nitrocellulose membrane (Schleicher and Schuell, Dassel, Germany) [60]. Membranes were probed with the rabbit polyclonal anti-human GALNT3 antibody (Sigma-Aldrich, Dorset, UK) followed by HRP-conjugated anti-rabbit IgG (Bio-Rad, Hemel Hempstead, UK) and ECL detection (GE Healthcare, Little Chalfont, UK). The membrane was stripped and re-probed with HRP-conjugated mouse anti-GAPDH antibody (Abcam, Cambridge, UK) as a loading control [55].

### In vivo Gene Expression Studies

Total RNA was isolated from kidneys using the RNeasy mini kit (Qiagen, Crawley, UK). For extraction from bones, femora were pulverised under liquid nitrogen, homogenised in QIAzol lysis reagent (Qiagen, Crawley, UK), mixed with chloroform and centrifuged at 12000 g for 15 min and 4°C. RNA was extracted from the upper aqueous phase using the RNeasy mini kit (Qiagen, Crawley, UK). Two micrograms of RNA was used to synthesize cDNA using AffinityScript multiple temperature reverse transcriptase (Agilent Technologies, Edinburgh, UK) according to the manufacturer’s instructions. cDNA templates were amplified by quantitative PCR using SYBR Green (Applied Biosystems, California, USA) and Applied Biosystems® 7500 Fast Real-Time PCR System [37], and gene-specific primers for *Fgf23*(F: CCACGGCAACATTTTTGGA, R: CTGGCGGAACTTGCAATTCT), *Galnt3* (F: TTCTCTGCACCGGGACCTT, R: GGCGGGCAGCGCTTA), *Klotho* (F:GGCTTTCCTCCTTTACCTGAAAA, R: CACATCCCACAGATAGACATTCG ), *Cyp27b1* (F: TGGAGTGGACACGGTATCCA, R: GGTCCCAGCTGTGATCTCAGA), *NPT2a* (F: CTCCTCCGGCTTGTTGGA, R: GACAGAGGTTCCGATGTTGGA), *NPT2c* (F: TTGCTTCAGAGCAGTAGTGTCTTCA, R: CCCCAACCCCCATGAGA) and *Gapdh* (F: AGCGAGACCCCACTAACATC, R: GGTTCACACCCATCACAAAC). Gene expression was assessed by SYBR green detection, normalized to expression of *Gapdh* and analyzed by the Comparative ΔΔC_T_ method to determine the difference in mutants relative to wild-type groups [37].

### Statistical Analysis

Mean values and standard deviations (SD) or standard errors of mean (SEM) were calculated and analysis performed using unpaired Student’s t-test for independent samples in which the Bonferroni correction for multiple testing was applied [54]. *P* values <0.05 were considered significant.
